# Chronic nonbacterial osteomyelitis: the role of whole-body MRI

**DOI:** 10.1186/s13244-022-01288-3

**Published:** 2022-09-16

**Authors:** Marcelo Astolfi Caetano Nico, Flávia Ferreira Araújo, Júlio Brandão Guimarães, Isabela Azevedo Nicodemos da Cruz, Flávio Duarte Silva, Bruno Cerretti Carneiro, Alípio Gomes Ormond Filho

**Affiliations:** Department of Musculoskeletal Radiology, Fleury Medicina e Saúde Higienópolis, Rua Mato Grosso 306, 1st Floor, Higienópolis, São Paulo, SP 01239-040 Brazil

**Keywords:** Autoinflammatory disease, Children, Chronic recurrent multifocal osteomyelitis, Whole-body magnetic resonance imaging

## Abstract

**Background:**

Chronic nonbacterial osteomyelitis (CNO), also known as chronic recurrent multifocal osteomyelitis, is a noninfectious autoinflammatory disorder that occurs primarily in children and adolescents and is characterized by episodic musculoskeletal pain with a protracted course.

**Main body:**

Traditionally, the diagnosis of CNO is made by exclusion and commonly requires bone biopsy to rule out infection and malignancy. However, bone biopsy may be avoided when imaging and clinical characteristic features are present, such as multifocal bone lesions at typical sites, no constitutional symptoms and no signs of infection in laboratory test results. Whole-body magnetic resonance imaging (WB-MRI) can assess signs of acute and chronic inflammation and enables the detection of CNO typical patterns of lesion location and distribution, thereby helping to exclude differential diagnosis. The goal of the present study paper is to review the main clinical and imaging aspects of the disease with emphasis on the role of WB-MRI in the diagnosis, assessment of disease burden and follow-up monitoring.

**Conclusion:**

Radiologists need to be familiar with the imaging features to suggest the diagnosis as the early therapy may help to avoid irreversible secondary damage of skeletal system.

## Key points

- CNO/CRMO is a chronic autoinflammatory syndrome occurring primarily in children and adolescents.
- Imaging plays a pivotal role in diagnosis, differential diagnosis and therapy monitoring.
- MRI/WB-MRI is the most sensitive imaging method to detect CNO lesions.

## Background

Chronic nonbacterial osteomyelitis (CNO) is a chronic autoinflammatory syndrome occurring primarily in children and adolescents, first reported by Giedion et al. in 1972 as “subacute and chronic symmetrical osteomyelitis” [1]. The peak incidence is between 7 and 12 years of age but can occur in all age groups [2]. Rarely, if disease onset occurs before 2–3 years of age, the possibility of monogenic autoinflammatory syndromes should be considered, such as familial chronic multifocal osteomyelitis (Majeed syndrome) and deficiency of the IL-1-receptor antagonist (DIRA), in addition to other features (i.e., dyserythropoietic anemia and early-onset pustular rash, respectively) [3]. 

The annual incidence of CNO is estimated at 0.4 per 100,000 children [4]. Although consistent epidemiological data are lacking, recent studies suggest that CNO is more common than previously established and may be one of the most prevalent autoinflammatory diseases [5, 6]. One of the reasons for the underdiagnosis is the relative lack of knowledge of this condition, which may lead to several segmental imaging evaluations of the affected joints and to invasive procedures such as bone biopsies, often delaying the diagnosis for years.

When the clinical picture is compatible and a characteristic lesion is found on initial imaging examination, whole-body magnetic resonance imaging (WB-MRI) is of fundamental importance in early diagnosis because it may assess both the symptomatic lesion that motivated the examination and possible asymptomatic lesions, confirming the multifocal character of the disease. In addition, WB-MRI provides the location and distribution of lesions demonstrating the typical pattern of symmetrical and bilateral involvement, along with disease activity data and possible complications.

In the present article, we review the epidemiological, etiologic, clinical and histopathological features of CNO and the typical patterns of bone involvement in MRI, highlighting the importance of WB-MRI in earlier diagnosis, therapeutic decision-making and follow-up.

## Pathophysiology

The pathophysiology of the disease remains not yet fully understood. The post-infectious origin suspected initially has been ruled out since no pathogen has been found and antibiotics have no effect on the disease course [7]. An autoimmune etiology has also been suggested, but no specific antibodies have been found [8]. However, recent findings from Hendrich et al. indicate that an imbalance between cytokines, with decreased expression of anti-inflammatory cytokines (IL-10 and IL-19) and increased expression of proinflammatory cytokines (IL-6, IL-20 and TNF-α), may be centrally involved in the molecular pathology of CNO. An imbalance in cytokines leads to accelerated osteoclast differentiation and activation, osteolysis and bone remodeling [9]. 

**Fig. 1:**
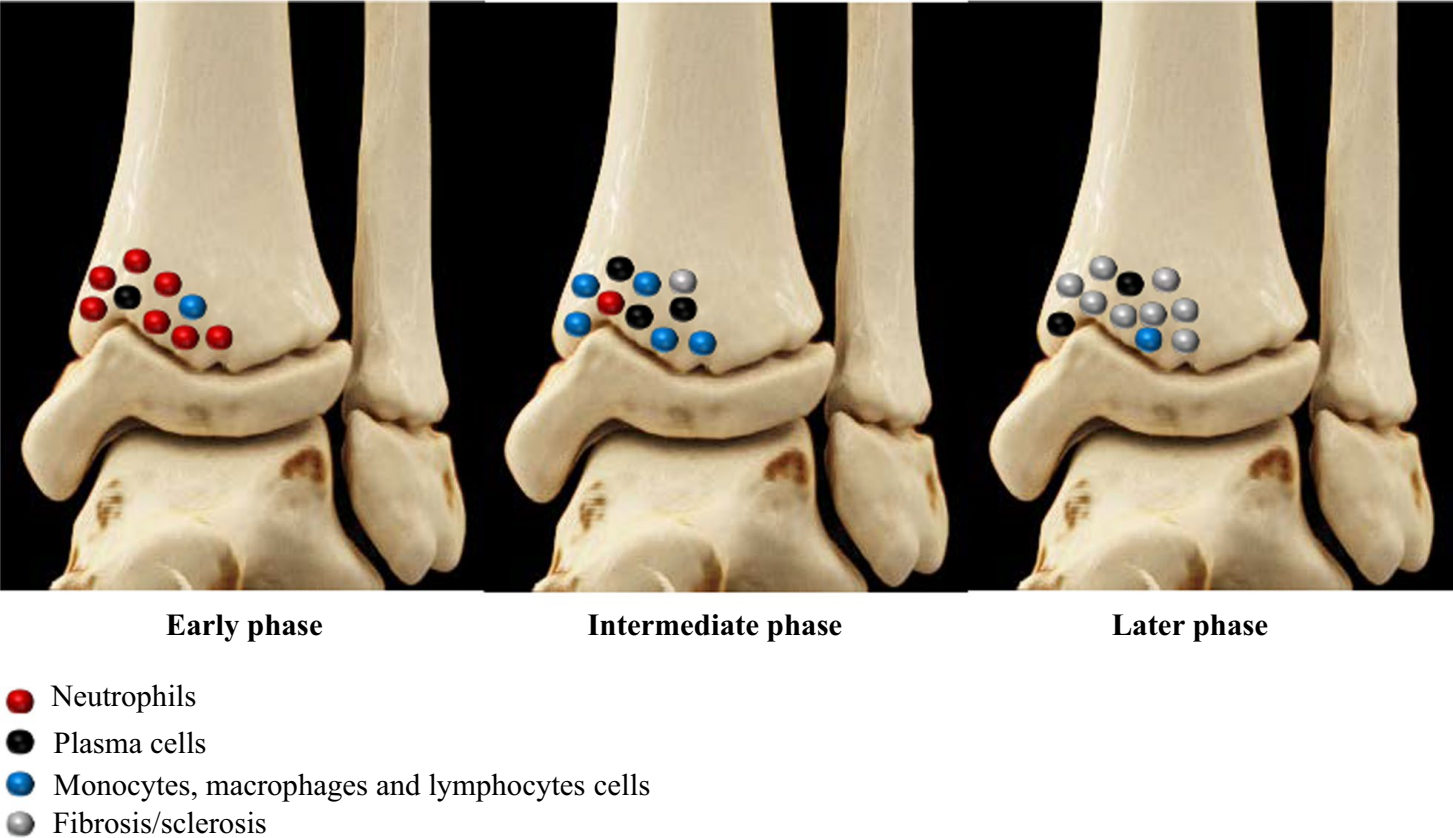
Schematic distribution of inflammatory cells related to the stage of CNO

In CNO, histological examination reveals nonspecific acute, subacute or chronic sterile osteomyelitis and cellular infiltrates are highly dependent on the disease stage. Early phase is characterized by a predominance of neutrophils and osteoclastic bone resorption. During later stages of the disease, monocytes, macrophages, lymphocytes and plasma cells can be detected, along with new bone formation (Fig. 1) [8, 9].

The distribution of CNO, specifically its tendency to involve the metaphyses and metaphyseal equivalents, is reminiscent of hematogenous osteomyelitis in children [10], but the reason why CNO is more common in these sites remains to be established.

## CNO and SAPHO syndrome

**Fig. 2:**
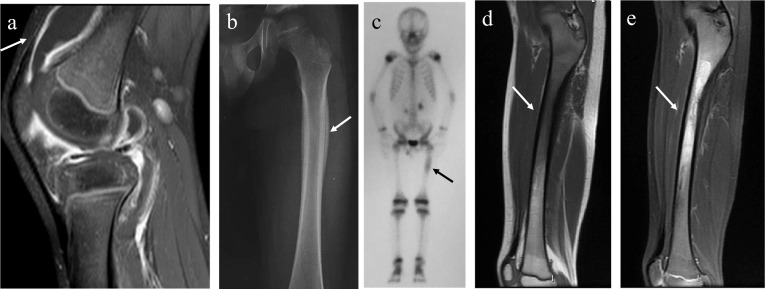
A 5-year-old boy presented with a swollen and painful right knee. Sagittal fat-saturated T1-weighted image (ceFST1WI) shows synovitis with joint effusion and inflammatory changes in adjacent soft tissues (**a**). After 5 months, he presented pain in his left thigh. Plain radiograph showed a single-layered periosteal reaction on the proximal diaphysis of the left femur (**b**) and bone scintigraphy shows uptake on this site (**c**). Sagittal T1WI (**d**) and fat saturated T2WI (FST2WI) (**e**) show edema and periosteal thickening of the proximal diaphysis of the femur. The diagnosis of chronic nonbacterial osteomyelitis was confirmed with histopathological nonspecific inflammatory changes and negative culture results from two bone biopsies. Synovitis occurs in 5–30% of cases of CNO and single lesions and diaphyseal involvement are rare forms of presentation (“SAPHO-like”)

Some authors have suggested that CNO and SAPHO (synovitis, acne, pustulosis, hyperostosis, and osteitis) lie along the same clinical spectrum and some believe that CNO is the pediatric presentation of SAPHO [11, 12]. In fact, CNO and SAPHO syndrome share numerous characteristics [12], including osteitis, unifocal or multifocal presentation, pustulosis, hyperostosis and a good general state of health (Fig. 2).

**Fig. 3:**
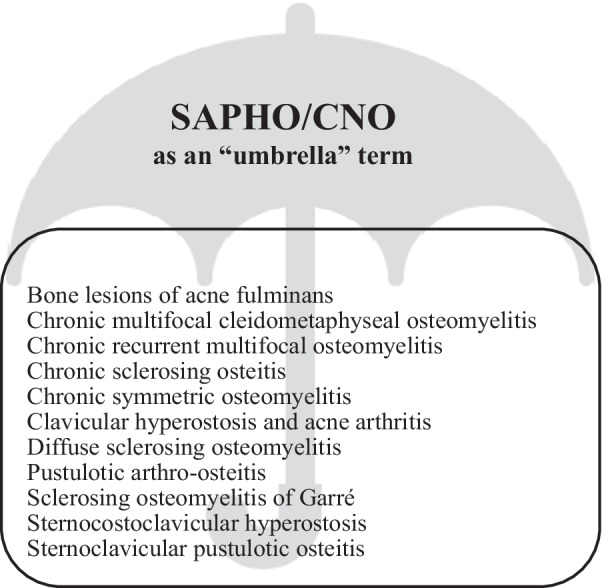
SAPHO/CNO as an “umbrella” term

The main differentiating clinical feature is the location of the inflammation. CNO predominantly affects the metaphysis of long bones and SAPHO affects the diaphysis and axial skeleton with involvement of the sternoclavicular and sternocostal joints [13]. Currently, the term SAPHO/CNO functions as an umbrella term covering many related clinical entities (Fig. 3).

## CNO and spondylarthritis

**Fig. 4:**
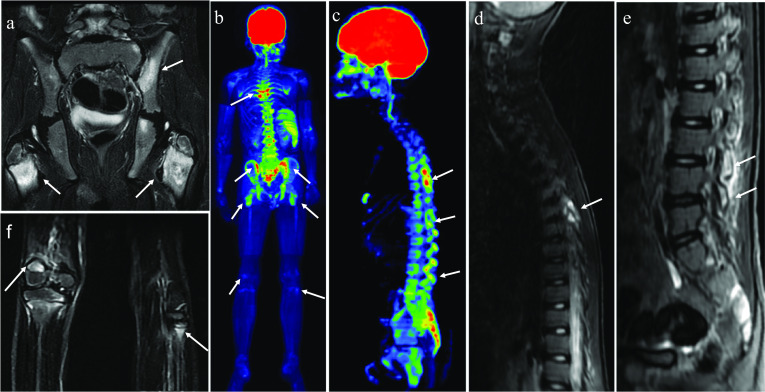
10-year-old child with CNO and a history of intermittent hip pain since he was 4 years old with elevated markers of inflammation under investigation for juvenile idiopathic arthritis. MRI of the pelvis showed bone marrow edema in the intertrochanteric periphyseal regions and in the sacroiliac joint (**a**), which was suggestive of CNO. WB-MRI was performed on the same day (**b**–**f**). Functional diffusion-weighted imaging (DWI) sequences (**b** and **c**) detected multiple lesions in the posterior elements of the thoracolumbar spine, sacroiliac joints and intertrochanteric regions, in addition to the distal metaphysis of the right femur and proximal metaphysis of the left fibula. The lesions are demonstrated in STIR morphological sequences as signal change with an edema pattern in the corresponding locations (**d**, **e** and **f**). HLA B27 was positive

CNO belongs to the family of autoinflammatory diseases with osseous manifestations. Some authors [14] consider CNO as an unusual form of spondyloarthropathy (SpA) because, in addition to osteitis, spondylarthritis-like features may be present, such as axial involvement, occurrence of inflammatory bowel disease (IBD) and an increased prevalence of the HLA-B27 (Fig. 4). However, CNO is an atypical SpA because of the absence of a familial background, the frequency of anterior thoracic involvement and the often unilateral sacroiliitic involvement [15].

A retrospective multicenter study with 41 children diagnosed with CNO and SAPHO syndrome found that osteomyelitis in the pelvic region is significantly associated with other features of juvenile spondylarthritis [14]. HLA-B27 was positive in 21% of the patients tested, which is lower than that in patients with enthesitis-related arthritis.

## Clinical features

The clinical spectrum and severity of CNO vary significantly, with asymptomatic/mildly symptomatic involvement of single bones at one end and chronically active recurrent multifocal osteitis at the other end, also referred to as CRMO [16, 17]. A large cohort study with 178 patients separated into three phenotypes (severe, mild and intermediate) revealed that the severe phenotype (all male patients) had the worst prognosis and was associated with an initial multifocal form of CRMO and inflammatory syndrome [18]. 

Clinical signs of bone inflammation include pain, tenderness or swelling. Other symptoms may be caused by paraosseous inflammation involving nerves, vessels, skin or synovia. The disease is marked by exacerbations and remissions, and symptoms may either recur in areas previously affected or involve new sites over time [10, 16].

Patients may experience concomitant systemic symptoms, such as low-grade fever (20%), malaise, weight loss and fatigue. The laboratory findings are nonspecific, with slight increases in serum inflammatory markers [C-reactive protein (CRP), erythrocyte sedimentation rate (ESR)], and a normal blood count [2, 19]. In a series including 75 children with CNO, the elevation of inflammatory markers significantly predicted the number of sites of CNO involvement, suggesting that the number of MRI sites may represent a marker for disease activity [20]. 

A subset of children with CNO may exhibit inflammatory organ involvement, including psoriasis in 2–17%, palmoplantar pustulosis in 3–20% and IBD in 3–7% of patients with CNO [2]. 

## Diagnostic imaging approach

There is no confirmatory diagnostic test for CNO. The diagnosis of CNO is traditionally one of exclusion of other diseases, such as acute bacterial osteomyelitis, bone tumors, blood diseases, fibrous dysplasia and histiocytosis [8]. Due to the gradual onset of the symptoms, minimal findings on clinical examination, relatively normal laboratory studies and lack of awareness of this condition, children with CNO may experience delays in diagnosis. These delays may lead to prolonged use of antibiotics, multiple surgeries, repeated bone biopsies and excessive radiation exposure. The median time between initial symptoms and diagnosis of CNO is 2 years [21], and 69% of the patients consulted with 2–5 physicians before receiving the final diagnosis [22]. It is common for the patient to undergo multiple segmental examinations of the joints involved over the time before the diagnosis of CNO is suggested.

Diagnostic imaging frequently includes conventional radiography and/or regional magnetic resonance imaging (MRI) at sites of pain. Plain radiography may reveal sclerotic, lytic or mixed lesion, according to the time of disease evolution. Hyperostosis and periosteal reaction are also suggestive features, though less frequently observed [8]. Characteristically, there is no abscess formation, fistula or sequestra, which are often identified in pyogenic osteomyelitis [19]. 

Occasionally, computed tomography (CT) may be useful when MRI is unavailable, but it should be avoided in children [23]. 

MRI is highly sensitive for detecting inflammatory lesions without radiation exposure, which is important, especially in young children, as MRI can reveal the site, features, and patterns of bone involvement characteristic of CNO [24]. On MRI, active disease exhibits edematous marrow changes, including T1 hypointensity and hyperintensity on both T2 and short time inversion recovery (STIR) sequences.

Therefore, it is important for radiologists to recognize the imaging characteristics of bone lesions in CNO and promptly suggest whole-body MRI in suspected cases, which may lead to a reduction in the time between the onset of symptoms and diagnosis [25]. In the past, 99 m-technetium bone scintigraphy was used, but should be considered obsolete and only be used if WB-MRI is unavailable.

**Fig. 5:**
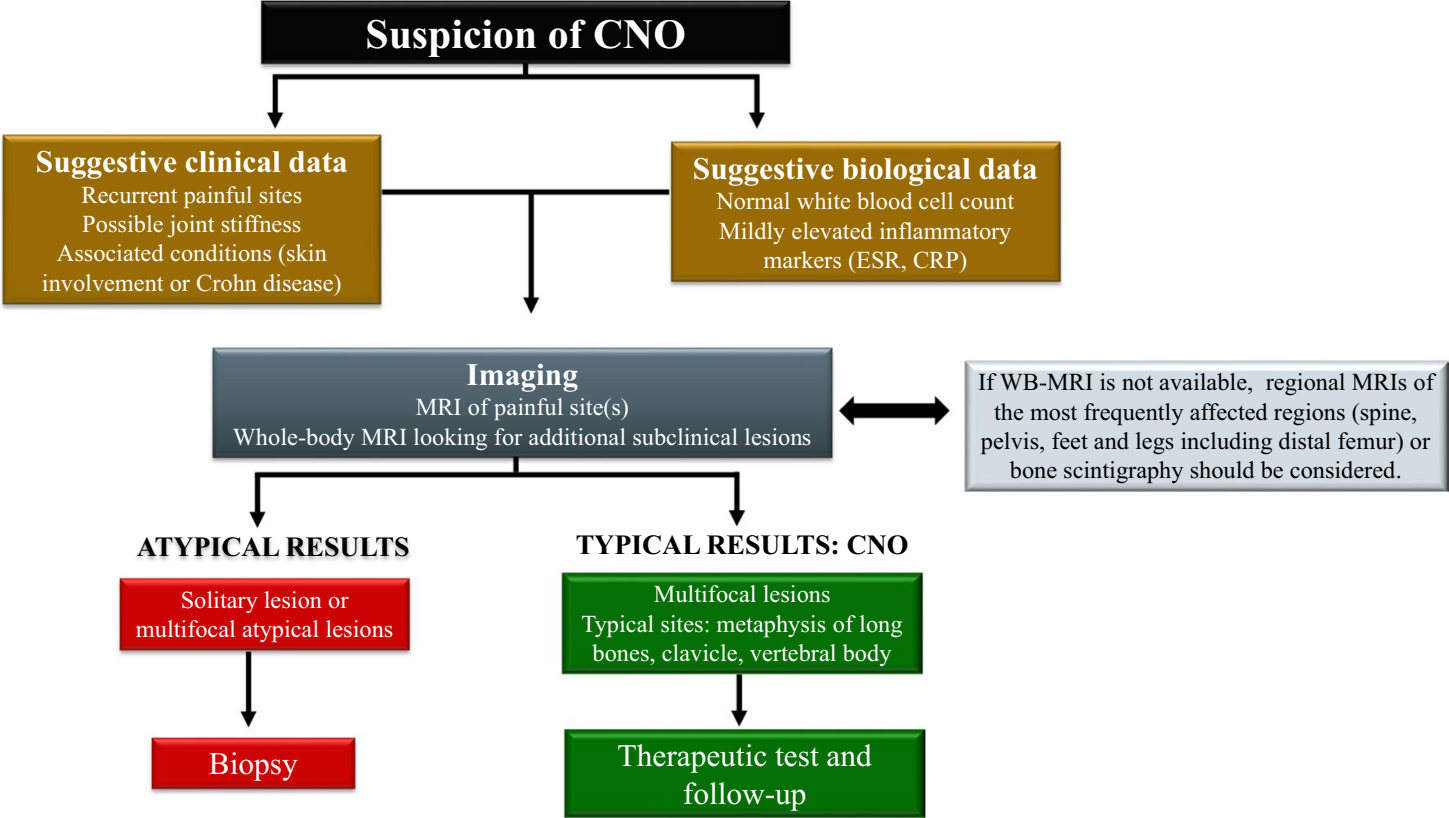
Diagnostic approach. Adapted from Falip et al. [8]

Most recently, some authors have suggested that biopsy could be avoided when there are characteristic findings, such as suggestive clinical data and multiple bone lesions at typical sites (clavicle, metaphysis of long bones, vertebral body) with normal laboratory test results and no constitutional symptoms [2, 8]. Therefore, bone biopsy is reserved for cases where the clinical or radiological findings are inconclusive or multifocality is not revealed (Fig. 5) [8].

### Imaging findings

The imaging aspects of CNO include multiple bone lesions, mainly affecting the metaphyses of long bones, clavicle, vertebrae and mandible [2]. Symmetric involvement is common and multifocality is virtually always present [19]. In 30% of cases, CNO involves the adjacent joint with the presence of synovial thickening and/or damage to the articular cartilage [26]. 

CNO is the most common non-neoplastic process involving the clavicle in children and adolescents, and clavicular involvement was noted in 38% of patients with CNO [27]. 

**Fig. 6:**
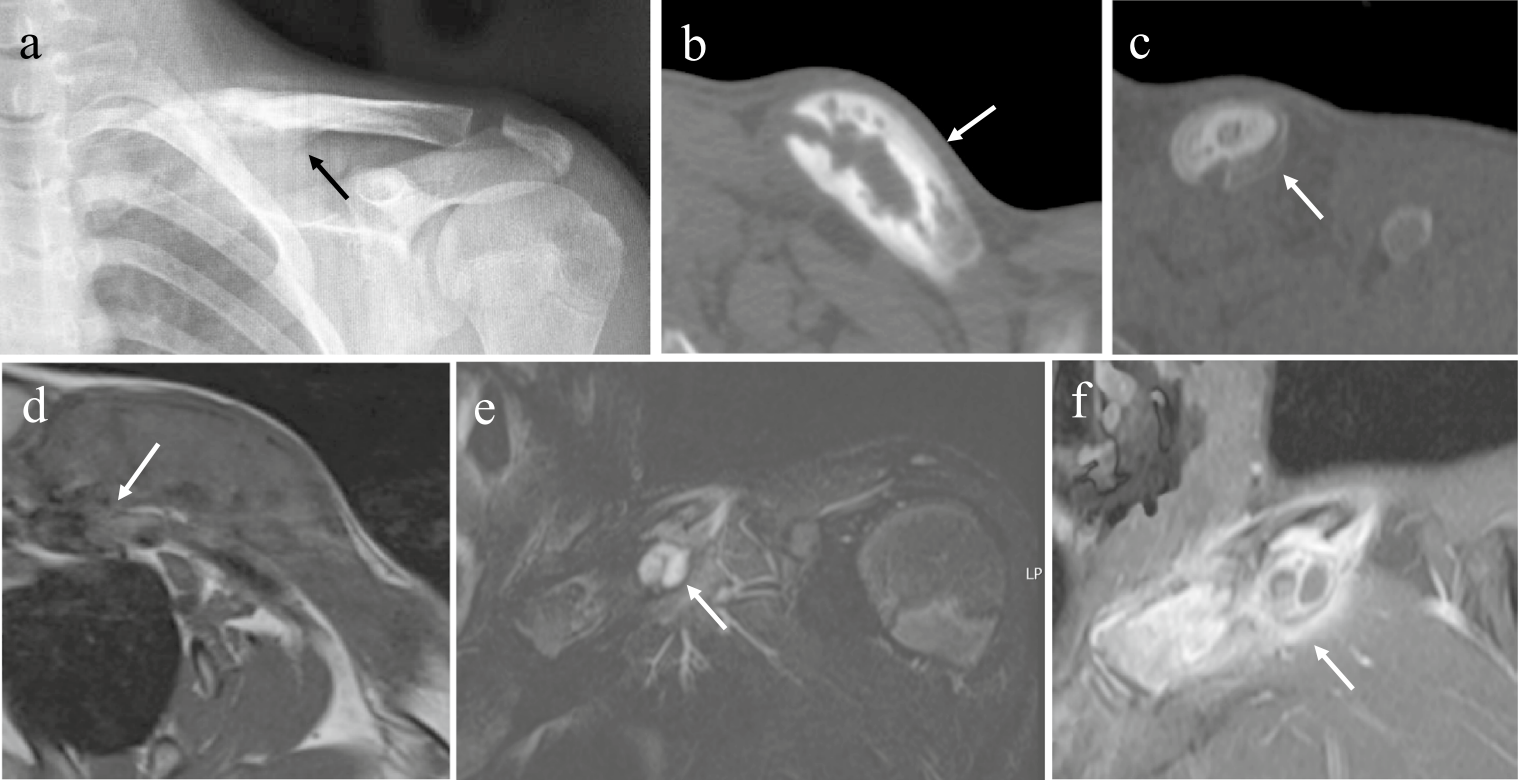
A 14-year-old boy with a 3-week history of pain and swelling of the left clavicle. Anteroposterior radiograph reveals hyperostosis, sclerosis and periosteal reaction of the medial third of the clavicle (**a**). CT scan illustrates better clavicle periosteal reaction and erosions (**b** and **c**). Axial T1WI (**d**), coronal FST2WI (**e**) and coronal ceFST1WI (**f**) images show the same findings, in addition to bone marrow edema and heterogeneous enhancement within the clavicle and surrounding soft tissues. Biopsy revealed sterile inflammation. CNO is the most common disease involving the medial third of the clavicle in all age groups

**Fig. 7:**
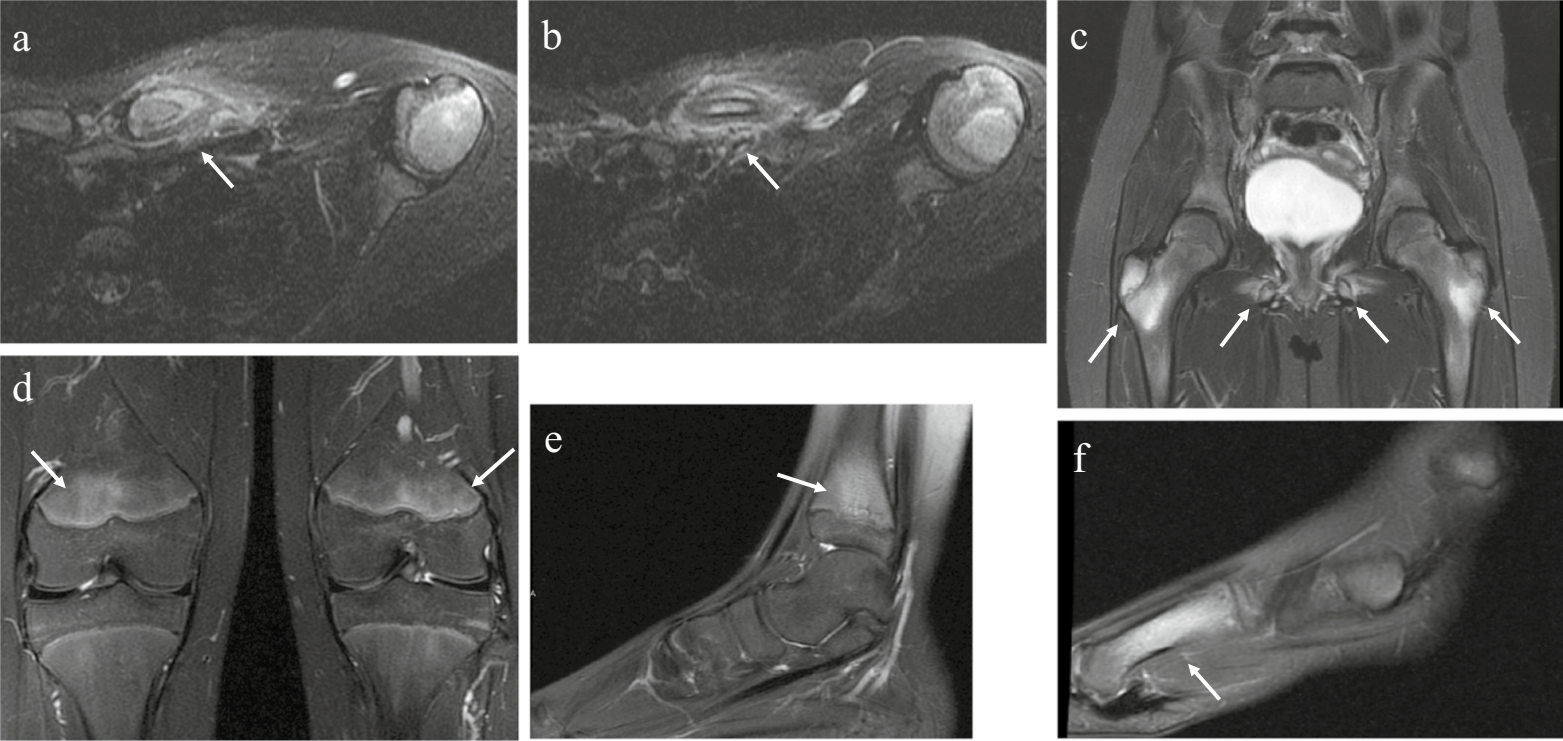
An 11-year-old girl with a 3-month history of pain of the left clavicle. The laboratory findings showed slight increases in serum inflammatory markers (ESR = 61 mm) and a normal blood count. Axial FST2wI images revealed bone marrow edema of medial third of the clavicle with periosteal reaction and soft tissue edema (**a** and **b**). A study of the hip (**c**), knees (**d**) and ankle (**e**) showed periphyseal bone marrow edema and diaphyseal edema of the first metatarsal of the left foot (**f**)

These lesions typically involve the medial third of the clavicle with periosteal reaction, hyperostosis and soft tissue signal abnormality (Fig. 6). Clavicle is an atypical location for bacterial osteomyelitis and such as the diagnosis of CNO should be considered in the differential diagnosis of expansive lesions affecting the medial third of the clavicle in this age group [10] (Fig. 7).

Spinal lesions were recently reported as a classic site and one of the most common sites for CNO [8, 27], but these lesions may be easily overlooked, as they do not necessarily cause pain [4]. Spinal lesions are usually multifocal and tend to involve noncontiguous vertebrae without crossing the disk, distinguishing it from infectious spondylodiscitis [27]. The thoracic portion of the spine is reportedly involved most often (78% of spinal sites) [8]. MRI shows an increased signal of vertebral marrow and endplate irregularity and can demonstrate vertebral height loss, which is the most common pathological fracture in CNO [10, 27]. 

**Fig. 8:**
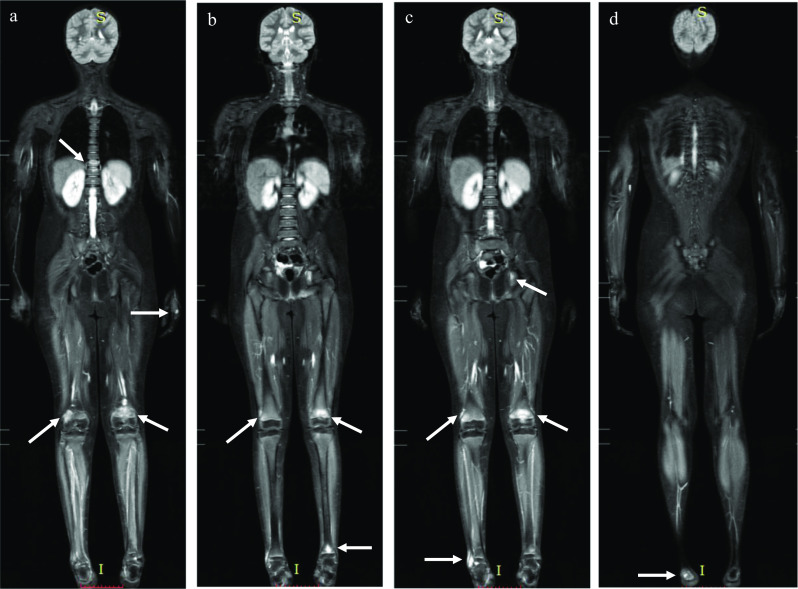
CNO in an 8-year-old girl complaining of hip and leg pain. Whole-body MRI (**a**–**d**) depicted multifocal bone lesions at the distal metaphysis of both femurs (**a**–**c**), distal metaphysis of the left tibia (**b**) and right fibula (**c**), right calcaneus (**d**), left acetabulum (**c**), left metacarpal bone head (**a**) and bone marrow edema at a mid-thoracic vertebral body (**a**). The vertebral lesion was asymptomatic and its detection in a preclinical scenario may change the treatment approach and prevent complications

The detection of spinal involvement is important for preventing pathological fracture, as vertebral height is not regained with treatment [27, 28]. The early detection of a spinal lesion at a subclinical stage may guide treatment and help to prevent complications (Fig. 8).

### WB-MRI protocol

Our protocol includes morphological sequences such as "short time inversion recovery" (STIR), sensitive for identifying foci of bone marrow edema, and T1-weighted sequences, useful for differentiating true bone lesions from those of normal bone marrow conversion. In addition, it combines functional sequences, such as diffusion-weighted imaging (DWI), which have high sensitivity in detecting foci of edema of the bone marrow, increasing the conspicuity in the identification of focal bone lesions. DWI may be useful to help distinguish between benign and malignant spine fractures and the possible complications of CNO [24, 26]. 

**Fig. 9:**
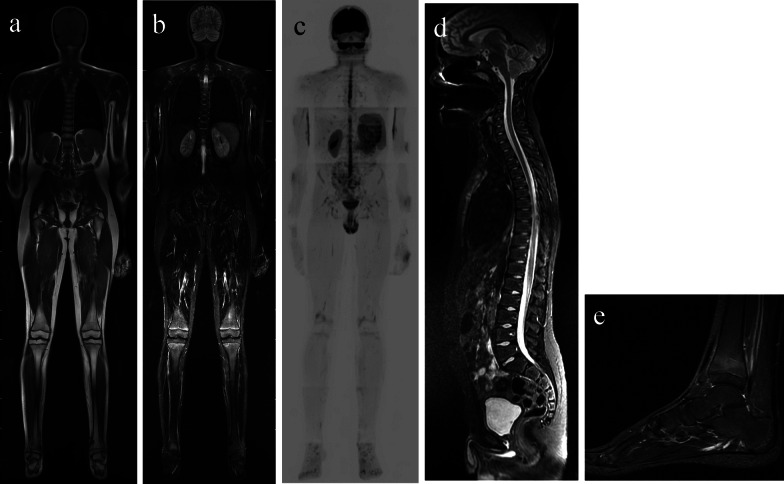
MRI protocol. Coronal T1 (**a**), coronal STIR (**b**), reconstructed coronal DWI inverted grayscale (**c**), sagittal STIR (**d**) of the whole spine and sagittal STIR of the feet (**e**)

WB-MRI protocols include coronal images of the entire body and sagittal images of the entire spine acquired with fluid-sensitive sequence (STIR, turbo-inversion recovery–magnitude, or fat saturation) without contrast, axial sequences of the pelvis and knees and sagittal images of the ankles and feet. Sagittal images of the spine are also included since spinal involvement is common and has important clinical and prognostic implications, offering the possibility of individualized treatment (Fig. 9).

WB-MRI with a scanning time of approximately 40 min is well tolerated even by children and achieves an adequate image quality in the vast majority of examinations [29]. 

### The role of WB-MRI in diagnosis

**Fig. 10:**
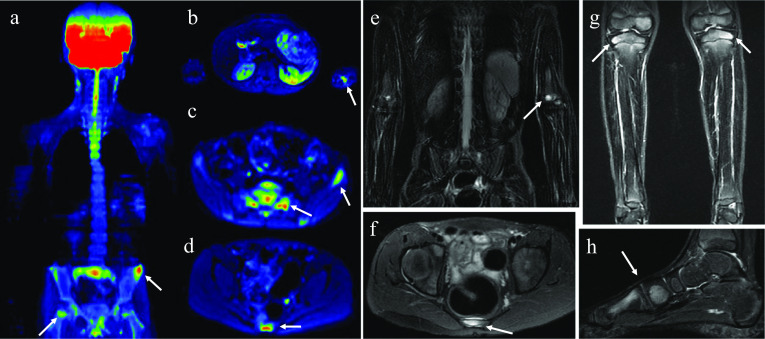
6-year-old male child with history of pain in his right hip and alteration of his gait for 6 months, with positive inflammatory tests, without trauma or fever and an investigation of hip synovitis. Initial MRI of the pelvis showed periphyseal lesions with symmetrical and bilateral distribution, in addition to involvement of the lower lumbar spine (not shown), raising the possibility of CNO. The WB-MRI study was suggested and performed 24 h after the first examination (**a**–**h**). DWI sequences (**a**–**d**) showing lesions already identified in the previous pelvic examination and detection of a new asymptomatic lesion in the capitellum (**b**), also demonstrated by the morphological sequence STIR (**e**). STIR sequences of the pelvis (**f**), legs (**g**) and left foot (**h**), with peripheral edema in the vertebrae, lateral tibial plateaus and in the medial cuneiform region and in the first metatarsal region, respectively. In WB-MRI, some lesions were detected in asymptomatic sites, demonstrating the multifocality and characteristic distribution of the disease and confirming its diagnosis

Whole-body MRI allows a wide coverage assessment of both the skeleton and soft tissues with high spatial resolution and is the gold standard imaging modality. It has the capacity to detect subclinical foci of bone marrow edema, demonstrating the multifocal involvement of CNO and the typical patterns of lesion locations and distribution, which helps in the diagnosis and potentially may avoid the need for biopsy (Fig. 10). In addition, WB-MRI can assess both disease activity and skeletal damage, allowing the assignment of the stage of CNO and depicting spinal lesions, thus offering patients a more individualized treatment plan. It is also useful for identifying complications and determining response to treatment [8, 24].

The suspicion of CNO with WB-MRI is higher when it is possible to recognize the typical patterns of bone involvement. The typical lesion in MRI is a hyperintense geographic metaphyseal lesion adjacent to the growth plates of the long bones of the lower extremities or additional lesions in the spine, pelvis, clavicle and/or sternum. In a study with 53 WB-MRIs of children and adolescents, 75% showed combinations of at least two of these typical MRI findings, which led to a higher suspicion of CNO [25]. 

**Fig. 11:**
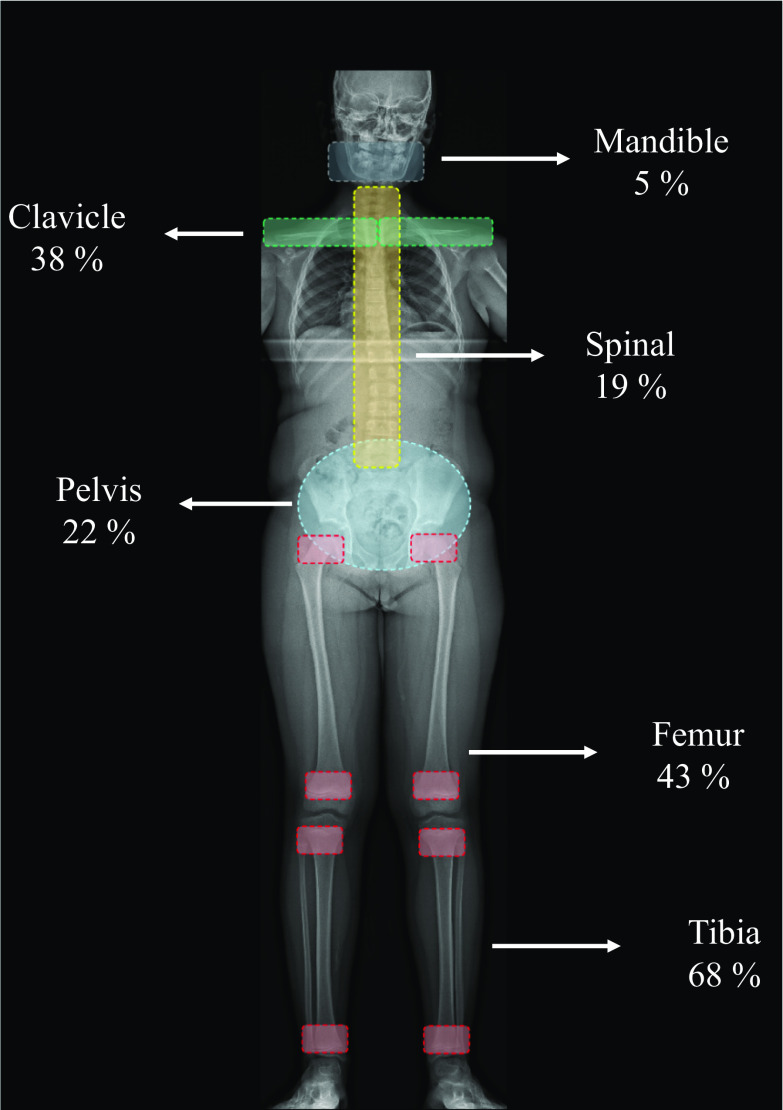
The most common affected bones in CNO and the percent of patients with each type of lesion site. Adapted from Andronikou [27]

A retrospective study performed by Andronikou et al., with 37 children diagnosed with CNO who underwent WB-MRI, identified 317 lesions in 20 different bones, with 89% of patients having multifocal disease. They noticed three patterns: (1) a tibioappendicular multifocal pattern, which is a multifocal appendicular lesion with predominantly tibial involvement; (2) a claviculospinal pauci-focal pattern, which is a primarily clavicular group with a paucity of other lesions predominating in the spine; and (3) a tibioclavicular crossover pattern, with synchronous clavicular and tibial involvement [27]. The most common sites of bone involvement and the percent of patients with each type of lesion site are summarized in.Fig. 11 [27].

**Fig. 12:**
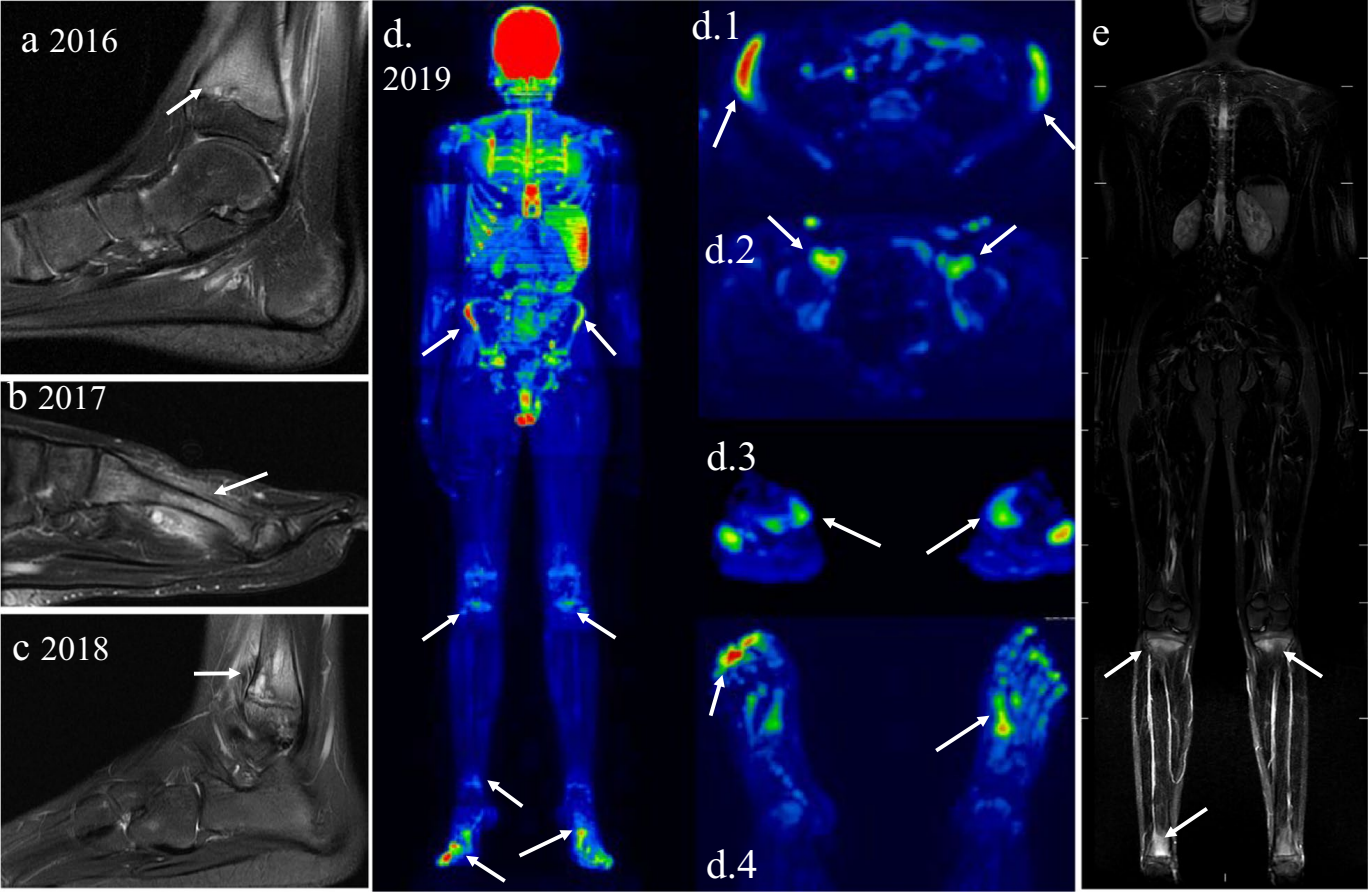
A 10-year-old boy who complained of pain in his left ankle and presented with a metaphyseal lesion in the distal tibia (**a**) which was initially suspected of bacterial osteomyelitis, despite negative biopsy and culture. In 2017, a foot examination showed a lesion in the second metatarsal of the left foot (**b**) and in the following year, lesions in the distal fibula were detected (**c**), which led to the hypothesis of CNO. WB-MRI was then performed and showed multiple lesions dispersed throughout the skeleton, in typical bilateral locations, confirming the diagnosis of CNO. WB-MRI DWI (**d**) and STIR (**e**) detected involvement of the iliac bones (d.1), anterior acetabulum column (d.2), bilateral tibial proximal metaphysis (d.3) and bones of the metatarsus (d.4) in correspondence with the STIR sequence of the whole body (**e**)

The diagnosis of CNO can be very challenging in some cases. Often, the diagnosis is only considered after several segmental evaluations of the symptomatic sites (Fig. 12).

### WB-MRI in follow-up, prognosis and therapeutic response

WB-MRI is useful to establish the disease burden of CNO by quantifying the number of lesions in the patient and the lesion extent (i.e., proportions of metaphyseal, epiphyseal and paraphyseal lesions) [27]. In a large study including 486 patients with CNO from 19 countries, the mean number of detected lesions by MRI was 4.1 per person [30]. 

**Fig. 13:**
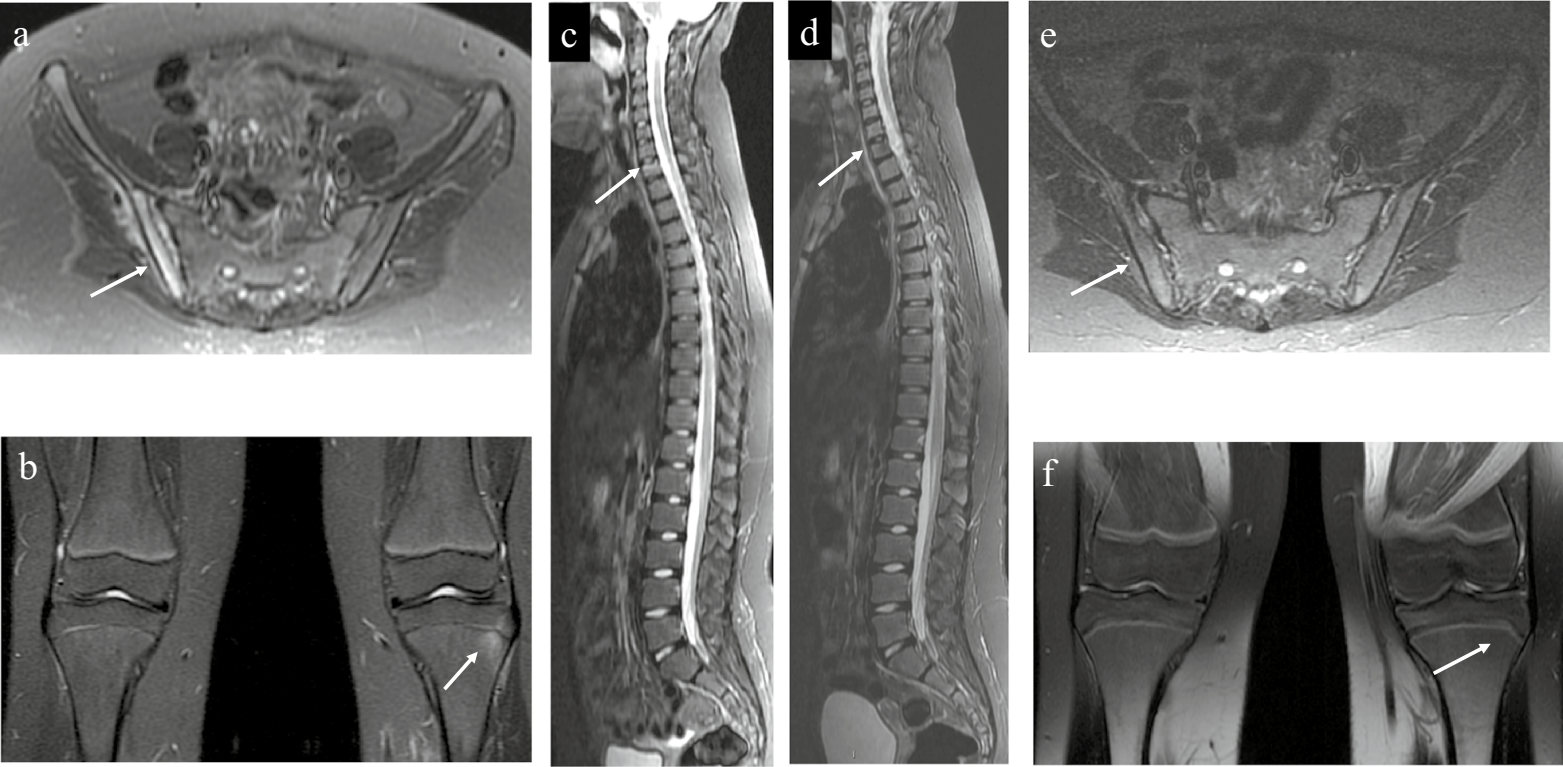
A 10-year-old girl complaining of thoracic and lumbar pain for two years who was initially treated for Langerhans cell histocytosis, despite two negative biopsies, without resolution of the pain. A WB-MRI was performed, showing bone marrow edema in the iliac portion of the right sacroiliac joint with joint effusion (**a**), lesion in the left proximal tibial metaphysis extending to the epiphysis (**b**) and edema and height loss of the vertebral body of T1 (**c**). The patient started treatment for CNO with non-steroidal anti-inflammatory drugs (NSAIDs) and pamidronate and showed clinical improvement. WB-MRI performed after 3 months showed stability of the T1 fracture (**d**) and complete resolution of the lesions of the pelvis (**e**) and the knee (**f**), without the appearance of new lesions

The number of radiological lesions has been used as a marker of disease activity in the context of the Pediatric CNO (PedCNO), developed by Beck et al. [31] The PedCNO score also includes ESR and severity of disease estimated by the physician, severity of disease estimated by the patient/parent and the childhood health assessment questionnaire (CHAQ) [31]. For such evaluation, it is important to perform WB-MRI at baseline and in the follow-up to help evaluate the therapeutic response [32] (Fig. 13). Complete resolution of lesions provides evidence of disease control [33].

A large cohort study performed by Arnoldi et al. was the first to directly correlate clinical findings of CNO and WB-MRI findings by creating a radiological index for CNO (RINBO). This scoring system uses WB-MRI as a tool to evaluate the number, size, extramedullary affection and spinal involvement of CNO. The goals of RINBO are to grade the intensity of disease and to simplify the evaluation of progression, stability or remission during the disease course. Additionally, RINBO aims to help monitor treatment response and improve therapeutic decisions [29]. 

## Treatment

**Fig. 14:**
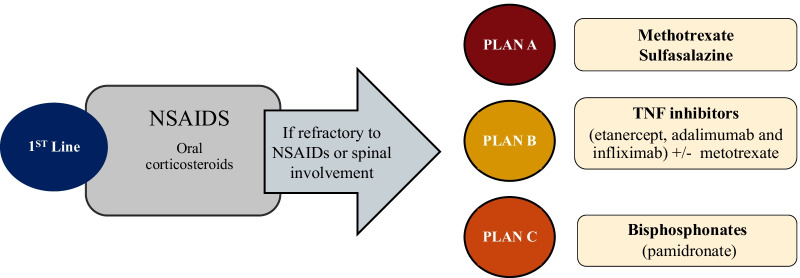
Treatment options of CNO

No consensus or guidelines exist for the treatment of CNO, and most evidence comes from small case series or retrospective cohorts. To our knowledge, no randomized controlled trials relating to CNO treatments have been published or are currently enrolling. Figure 14 summarizes the most common treatment options [34, 35].

CNO has an unpredictable course, although most cases undergo spontaneous resolution within several months to several years.

CNO of long bones can result in orthopedic complications such as limb-length discrepancy, which is the most common physical deformity, fracture, thoracic kyphosis secondary to vertebral body compression and physeal bars [10].

## Conclusion

CNO is a diagnosis of exclusion and is often delayed. It has high variability in presentation, sites, recurrence rates and prognosis. The typical clinical presentation is a child or adolescent with a history of chronic multifocal bone pain, usually with a normal blood count and mildly elevated inflammatory markers. The characteristic lesions are metaphyseal, multiple and symmetric, involving the lower extremities, pelvis, spine, clavicle, and mandible.

Currently, WB-MRI is the most sensitive imaging method to detect asymptomatic and radiographically occult multifocal lesions, demonstrating the multifocal pattern of the CNO and characteristic lesion distribution, thus helping to establish the diagnosis and reducing the need for invasive diagnostic procedures. Radiologists need to be familiar with the imaging features to suggest the diagnosis.

## Data Availability

All available material is included in this manuscript.

## Abbreviations

ceFST1WI: Fat-saturated T1-weighted image
CHAQ: Childhood health assessment questionnaire
CNO: Chronic nonbacterial osteomyelitis
CRMO: Chronic recurrent multifocal osteomyelitis
CT: Computed tomography
DIRA: Deficiency of the IL-1-receptor antagonist
DWI: Diffusion-weighted imaging
IBD: Inflammatory bowel disease
MRI: Magnetic resonance imaging
NSAIDs: Non-steroidal anti-inflammatory drugs
SAPHO: Synovitis, acne, pustulosis, hyperostosis and osteitis
SpA: Spondyloarthropathy
STIR: Short time inversion recovery
T1WI: T1-weighted imaging
T2WI: T2-weighted imaging
WB-MRI: Whole-body magnetic resonance imaging
